# Spontaneous coronary artery dissection causing acute myocardial infarction in a 62-year-old postmenopausal woman without co-morbidities: a case report

**DOI:** 10.1186/1752-1947-6-430

**Published:** 2012-12-28

**Authors:** Sobia Mujtaba, Vankeepuram S Srinivas, Cynthia C Taub

**Affiliations:** 1 Jacobi Medical Center/Albert Einstein College of Medicine, 1400 Pelham Parkway South, Building #1 (3N-21), Bronx, NY, 10461-1138, USA; 2 Jack D Weiler Hospital of the Albert Einstein College of Medicine, 1825 Eastchester Road, Bronx, NY, 10461-2372, USA

## Abstract

**Introduction:**

Spontaneous coronary artery dissection is an important yet rare cause of acute coronary syndrome. The available literature shows a higher risk factor for women, notably during pregnancy and puerperium. The incidence in postmenopausal women is exceedingly rare, and is more commonly seen in association with concurrent predisposing factors.

We illustrate an extremely rare case of a 62-year-old post-menopausal woman presenting with an acute myocardial infarction secondary to spontaneous dissection of the left anterior descending artery. Subsequent investigations did not reveal the presence of any co-morbidities.

To the best of our knowledge, our patient is one of the oldest documented cases of spontaneous coronary artery dissection on record, and is notable for having no known underlying risk factors for development of spontaneous coronary artery dissection.

Given the paucity of literature on spontaneous coronary artery dissection, particularly in postmenopausal women, we believe this case will provide further insight into the clinical presentation and management of this rare entity.

**Case presentation:**

A 62-year-old previously healthy postmenopausal Hispanic woman presented with chest pain and was found to have an ST elevation myocardial infarction. Cardiac catheterization revealed a dissection in her left anterior descending artery. Revascularization was deferred; our patient received appropriate medical management and remained asymptomatic. A full panel of tests was done to exclude underlying connective tissue disorders and vasculitis. On subsequent follow-up, our patient continued to do well and all work-up was reported as negative.

**Conclusion:**

We describe the varied presentation and subsequent management of a case of spontaneous coronary artery dissection and highlight the importance of considering spontaneous coronary artery dissection as a differential diagnosis even in older, postmenopausal women.

The consequences of a delay in diagnosis and appropriate management are associated with a high mortality and morbidity; hence we believe that reporting all cases of spontaneous coronary artery dissection, particularly in postmenopausal women, will add invaluable information to the limited literature on this rare condition.

## Introduction

Spontaneous coronary artery dissection (SCAD) is a rare entity; the overall incidence on coronary angiographies is around 0.2% [1]. The mean age of presentation is 42 years, with three-quarters of cases reported in women, of which 30% are peripartum [1].

Coronary atherosclerosis and the peripartum period are most commonly associated with the development of SCAD [2]. Other predisposing factors that have been reported include connective tissue disorders (Marfan’s syndrome, Ehlers-Danlos syndrome), vasculitis (for example, polyarteritis nodosa, systemic lupus erythematosus and eosinophilic arteritis), antiphospholipid antibody syndrome and inflammatory bowel disease [2]. Conditions associated with coronary shear, such as severe systolic hypertension, cocaine use and strenuous exercise, have also been linked to the development of SCAD [1]. However, the precise etiopathogenesis of SCAD remains unclear.

The clinical presentation of SCAD depends largely on the degree of compromise of myocardial blood flow, with most patients presenting with an acute coronary syndrome [1]. Undiagnosed or untreated cases may present in extremis with tamponade, cardiogenic shock or even sudden cardiac death [1].

A review of the literature is remarkable for only a few reported cases of SCAD in postmenopausal women; its incidence in this particular subset of patients, in the absence of known predisposing factors, is exceedingly rare.

We illustrate below the rare case of a 62-year-old healthy postmenopausal woman who presented with an acute myocardial infarction secondary to SCAD involving her left anterior descending artery, followed by a brief review of the literature on SCAD including the pathogenesis, treatment and prognosis.

## Case presentation

A 62-year-old previously healthy postmenopausal Hispanic woman without prior cardiac history presented to our emergency department with recurrent retrosternal chest pain. Our patient denied any illicit drug use, smoking or excessive alcohol consumption. She also denied any family history of heart disease. Of note, our patient reported that she had attained menopause around the age of 50 years; between the ages of 20 and 30 years, she had three full-term pregnancies resulting in the birth of her three children.

**Figure 1:**
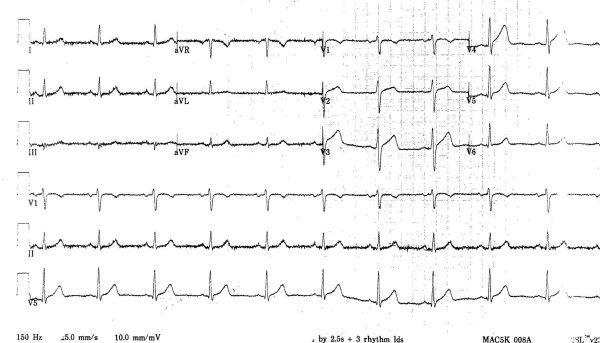
12-lead electrocardiogram showing ST elevations in the antero-lateral leads V2 to V5.

**Figure 2:**
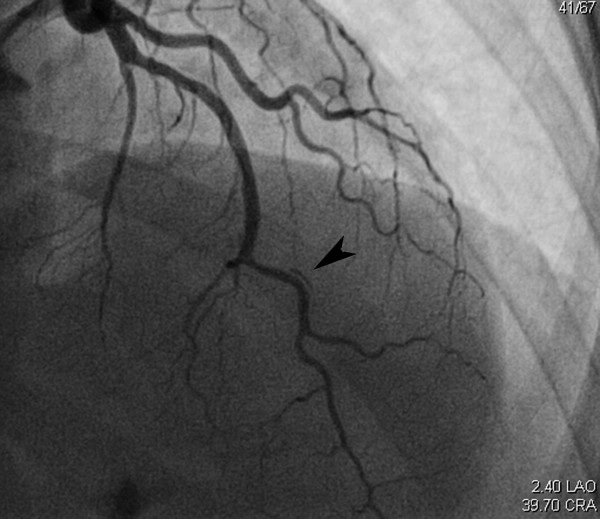
Spontaneous dissection of the mid portion of the left anterior descending artery (arrow).

An initial electrocardiogram was remarkable for ST elevations in the precordial leads (Figure 1), with a troponin I level of 0.02ng/ml. Her symptoms improved after receiving aspirin, clopidogrel, nitroglycerine, heparin, beta blockade and a statin. Repeat electrocardiograms after admission were remarkable for complete resolution of the ST segment elevation and our patient reported no further episodes of chest pain. Cardiac catheterization was performed which showed non-obstructive single vessel coronary artery disease with dissection in the mid portion of her left anterior descending artery, with significant luminal compromise (Figure 2). Intracoronary nitroglycerine was not given and no other tests were done to induce vasospasm. Because our patient was symptom free and demonstrated complete resolution of ST segment elevation, and there was no evidence of compromise in coronary flow, a decision was made to defer any revascularization and continue with medical management, including the addition of nifedipine. A full panel of tests, including erythrocyte sedimentation rate, C- reactive protein level, complement level, anti-nuclear antibody test, rheumatoid factor level, perinuclear anti-neutrophil cytoplasmic antibody and centrally accentuated anti-neutrophil cytoplasmic antibody tests, were found to be normal. Our patient continued to remain asymptomatic at two- and six-week follow-ups.

## Discussion

Since it was first reported in 1931 [3], only 220 cases of SCAD have been documented in the literature, likely an underestimate because 60% to 80% of cases present as sudden cardiac death [4].

SCAD has been reported predominantly in young women, particularly during pregnancy and puerperium. The incidence in postmenopausal women is rare; a review of the existing literature is notable for only three other cases of SCAD in postmenopausal women above the age of 60 years [5]. Of the three, one had diabetes and rheumatoid arthritis and one had severe systolic hypertension. The third women had no identifiable predisposing factors.

Our patient was postmenopausal, without any associated significant atherosclerotic disease, severe hypertension or other co-morbidities; and at 62 years of age, our patient is one of the oldest documented cases of SCAD on record.

The etiology of SCAD is likely multifactorial, the common insult being the weakening of the arterial wall, predisposing it to dissection. The proposed pathophysiologic mechanisms include inflammation and rupture of atherosclerotic plaques, enzymatic lysis of the arterial wall by peri-adventitial eosinophilic infiltrates in non-atherosclerotic vessels, changes in hormonal milieu associated with pregnancy and puerperium, presence of underlying vascular and connective tissue disorder, and mechanical weakening of the arterial wall caused by shear and stress associated with physical activity and cocaine use [2].

In atherosclerotic arteries, plaque inflammation and rupture can cause disruption of the intimal medial junction resulting in an intimal flap and subsequent intramural hematoma formation [3]. Coronary artery spasm vasospasm has also been thought to result in SCAD, however it remains unclear whether the vasospasm leads to dissection or the dissection itself leads to secondary vasospasm [2]. In the case of our patient, coronary angiography was remarkable for a single, non-critical proximal atherosclerotic lesion, with no impairment in coronary flow, followed by dissection with luminal compromise. It is our hypothesis that in this particular patient, the culprit lesion leading to ischemic symptoms and the ST elevation seen at the time of presentation was likely the result of a primary spontaneous dissection of the left anterior descending artery associated with secondary vasospasm.

Clinical presentations can span the clinical spectrum from unstable angina, to myocardial infarction and sudden cardiac death [2]. There has been an increasing recognition of cases due to the increasing use and availability of imaging modalities including angiography, intravascular ultrasound and computed tomography angiography [2]. Coronary angiography is considered the diagnostic test of choice [1]. On angiography, the typical appearance is that of a radiolucent line that represents the intimal medial flap, separating the flow between the true and false lumens. However, an intimal flap may or may not be visualized [4].

The literature on SCAD largely consists of case reports and case series; in the absence of clinical trials and guidelines, treatment decisions continue to be a challenge. Regardless of the management employed, the primary concern is to maintain the patency of the true lumen [1]. For patients presenting with an acute coronary syndrome, initial medical therapy should include antiplatelet and anti-ischemic agents along with anticoagulation with heparin [2]. If the suspicion for coronary artery dissection is high, fibrinolytics should be avoided. This can be challenging in the postmenopausal setting where the most common cause of atherothrombosis continues to be atherosclerotic coronary artery disease. Fibrinolytics, although they may help re-establish anterograde flow in the true lumen [4], carry the risk of increasing flow into the false lumen and propagating the dissection [1]. In patients with ongoing ischemia, revascularization with percutaneous coronary intervention or a coronary artery bypass graft should be undertaken; the decision to opt for either is largely based on the extent and location of the dissection [1]. After the resolution of the acute phase, a search into the etiology of the dissection should be pursued to guide future management. In addition to aspirin and beta blockers, patients with underlying coronary artery disease should receive cholesterol-lowering medication, whereas those who are at a risk for or who have underlying coronary vasospasm should be started on a calcium channel blocker [2]. Patients with SCAD during pregnancy or the peripartum period should be counseled on the increased risk of dissection associated with increasing parity and age [2].

Possible late complications include progression of the dissection and formation of pseudoaneurysms [1]. Patients should be followed for any symptoms of recurrent ischemia [2]. Stress testing with nuclear perfusion imaging is preferred over coronary angiography as a means of surveillance [2].

The overall survival for patients with SCAD has been reported as around 90%, with no significant difference in outcome being reported regardless of the treatment modalities employed [2].

## Conclusion

SCAD is a fascinating clinical entity. Although our case report is not the first of its kind, it does bring forward an extraordinarily uncommon presentation of a rare disease process.

Our patient’s age, absence of significant cardiac risk factors and lack of identifiable connective tissue disorder or vasculitis make her a rare case of SCAD; the etiology of the dissection remains a medical enigma.

Given the rare incidence of SCAD in postmenopausal women, there is a relative dearth of information regarding the clinical presentation, diagnosis and management in postmenopausal women. We hope that this case report and the associated literature review will serve to provide valuable insight into this rare yet intriguing disease process.

## Consent

Written informed consent was obtained from the patient for publication of this case report and any accompanying images. A copy of the written consent is available for review by the Editor-in-Chief of this journal.

## Competing interests

The authors declare that they have no competing interests.

## Authors’ contributions

CT is the consultant cardiologist responsible for the medical care of the patient, and read and edited the manuscript. VS provided the images, and read and approved the manuscript. SM completed the literature review and wrote the manuscript. All authors read and approved the final manuscript.
